# A simple technique to improve calculated skin dose accuracy in a commercial treatment planning system

**DOI:** 10.1002/acm2.12275

**Published:** 2018-02-06

**Authors:** Lilie Wang, Anthony J. Cmelak, George X. Ding

**Affiliations:** ^1^ Department of Radiation Oncology Vanderbilt University Medical Center Nashville TN 37232 USA

**Keywords:** Eclipse AAA, model‐based dose calculation, Monte Carlo calculations, skin dose

## Abstract

Radiation dermatitis during radiotherapy is correlated with skin dose and is a common clinical problem for head and neck and thoracic cancer patients. Therefore, accurate prediction of skin dose during treatment planning is clinically important. The objective of this study is to evaluate the accuracy of skin dose calculated by a commercial treatment planning system (TPS). We evaluated the accuracy of skin dose calculations by the anisotropic analytical algorithm (AAA) implemented in Varian Eclipse (V.11) system. Skin dose is calculated as mean dose to a contoured structure of 0.5 cm thickness from the surface. The EGSnrc Monte Carlo (MC) simulations are utilized for the evaluation. The 6, 10 and 15 MV photon beams investigated are from a Varian TrueBeam linear accelerator. The accuracy of the MC dose calculations was validated by phantom measurements with optically stimulated luminescence detectors. The calculation accuracy of patient skin doses is studied by using CT based radiotherapy treatment plans including 3D conformal, static gantry IMRT, and VMAT treatment techniques. Results show the Varian Eclipse system underestimates skin doses by up to 14% of prescription dose for the patients studied when external body contour starts at the patient's skin. The external body contour is used in a treatment planning system to calculate dose distributions. The calculation accuracy of skin dose with Eclipse can be considerably improved to within 4% of target dose by extending the external body contour by 1 to 2 cm from the patient's skin. Dose delivered to deeper target volumes or organs at risk are not affected. Although Eclipse treatment planning system has its limitations in predicting patient skin dose, this study shows the calculation accuracy can be considerably improved to an acceptable level by extending the external body contour without affecting the dose calculation accuracy to the treatment target and internal organs at risk. This is achieved by moving the calculation entry point away from the skin.

## 1 INTRODUCTION

Skin dose and its resultant toxicity, radiation dermatitis, has long been a concern of the radiation oncologist and is often a dose limiting toxicity of high‐dose treatments, particularly in head‐and‐neck and thoracic cancer patients.^1^, ^2^, ^3^, ^4^, ^5^ Improvements in radiation therapy treatment technique and immobilization devices reduce patient setup uncertainty but have exacerbated this clinical dilemma. It is well known that immobilization devices have a noteworthy deleterious effect on patient skin dose.^6^, ^7^, ^8^, ^9^ Therefore, accuracy of predicting skin dose by commercial treatment planning systems (TPS) is critical, as skin dose toxicity has a major impact on how well a patient tolerates treatment. It is known that model based dose calculation algorithms have limitations at the buildup region where the charge particle equilibrium (CPE) is not established. Therefore, accurate skin dose calculations would greatly help clinicians make appropriate treatment plan decisions for these patients where skin toxicity has historically been an issue.^10^

A number of investigations have focused on the accuracy of skin dose or entrance dose (or surface dose as sometimes referred) calculations in commercial TPS.^11^, ^12^, ^13^, ^14^, ^15^, ^16^, ^17^ Court et al.^11^ studied pencil beam convolution (PBC) algorithms in the Varian (Varian Medical Systems, Palo Alto, CA, USA) Eclipse system by comparing measured entrance dose to the Eclipse predicted dose. Oinam and Singh^15^ measured entrance dose in a phantom for a seven field 6 MV energy IMRT case and compared to the calculated dose by PBC algorithm and anisotropic analytical algorithm (AAA) in Eclipse version 8.6. Their results showed that AAA was more accurate than PBC, but both have limitations on predicting entrance dose accurately at depth less than 0.2 cm. Panettieri et al.^12^ compared entrance dose calculated by the PENELOPE system^18^ that calculated using PBC and AAA in Eclipse version 8.0. Similar results were observed in their study for tangential breast patients. Most of reported studies measure the entrance dose with various detectors and compare it to calculations by TPS software. All of these studies are based on phantom measurements and not by real CT based patient treatment planning. Results vary widely partly because of different TPS systems utilized, as well as the uncertainties in measuring entrance or skin dose accurately, especially in buildup regions.^13^, ^15^

With the recent advance in treatment delivery techniques, such as Volumetric Modulated Arc Therapy (VMAT), which may potentially reduce the skin dose toxicity compared with multiple static IMRT fields delivery techniques,^19^ it has become more important to know the accuracy of skin dose predicted by a commercial TPS in selecting treatment delivery techniques. The purpose of the study is to evaluate the accuracy of the skin dose calculated by a current Eclipse system (version 11) for real patient CT‐based treatment plans in treating various cancer sites. More specifically, the objective is focused on evaluating AAA dose calculation algorithm by using experimentally validated Monte Carlo simulations for photon beams from Varian TrueBeam accelerator utilizing different beam delivery techniques.

## 2 METHODS AND MATERIALS

### 2.A Photon beams and the commercial TPS studied

The commercial TPS evaluated in this study is Varian Eclipse Version 11.0 (Varian Medical Systems, Palo Alto, CA, USA) with the model‐based AAA dose calculation algorithm. The 6, 10, and 15 MV photon beams from a Varian TrueBeam accelerator are used in the study. The heterogeneity correction is employed in all AAA dose calculations; and, unless otherwise specified, the calculation grid size is 2.5 mm, which is a typical size in clinical practice. Although the grid size may affect the skin dose calculation accuracy, switching from 2.5 mm grid size to 1 mm grid size, which is smallest for AAA in Eclipse, only slightly improves the accuracy.^20^ In addition, the calculation time is much longer with 1 mm grid size, making it clinically unattractive. Furthermore, the grid size for MC dose calculations in this study (see below) is also set at 2.5 mm, which makes the comparison meaningful and justified. To avoid confusion, the skin dose is defined in this study as the mean dose to the skin structure of 5 mm thickness for the CT based dose calculations. To quantify the skin dose, the skin was contoured to be an area of 2 × 2 cm^2^, corresponding to a volume of about 2 cm^3^, which is of clinical interest. The skin dose predicted by Eclipse is compared with that of MC calculations which are benchmarked by measurements in phantoms. The term “entrance dose” is used for the phantom measurements.

### 2.B Monte Carlo simulations

The MC simulation code used in this study is the EGSnrc^21^ code and its user codes BEAMnrc^22^, ^23^ and DOSXYZnrc.^24^ The modulated realistic beams from the Varian TrueBeam accelerator with a Millennium 120 multileaf collimator (MLC) have been simulated by using BEAMnrc/DOSXYZnrc codes and calculated dose distributions have been validated.^25^, ^26^, ^27^

Varian TrueBeam phase‐space files^28^ (version 2.0) are used as the radiation sources at the plane before entering the secondary collimators, or jaws, and MLC. The jaw openings and MLC modulations are modeled in detail in the simulations as described in the study by Lobo and Popescu.^27^ The typical source phase‐space file for each beam energy used for simulation is about 20 GB in size containing about 900 million particles. The large number of particles used is necessary to achieve a statistical uncertainty of about 1% for MC calculations with a calculation grid size of 2.5 mm, which is the same as in Eclipse calculations. The EXACT boundary crossing algorithm is used and the electron cutoff energy (ECUT) is set at 0.7 MeV.

### 2.C Measurements

MC calculations have to be validated by measurements if they are used as a benchmarking tool. The EGSnrc MC code used in this study has been validated before on entrance dose calculations.^2^, ^29^ A further experimental validation was performed in this study where the MC calculated entrance dose was compared to that of phantom measurements using the nanoDot™ detector (Landauer Inc., Glenwood, IL, USA), the optically stimulated luminescence (OSL) dosimeters, which were pre‐screened and have a measurement uncertainty of 3–5% in this study. A well‐controlled geometry in which four solid water slabs of size 30 × 30 × 5 cm^3^ were stacked together to form a 30 × 30 × 20 cm^3^ phantom validated the accuracy of MC simulations. Photon beams of energies 6, 10, and 15 MV from a TrueBeam unit were delivered from lateral side at a gantry angle of 90 degrees, with a beam size of 4 × 4 cm^2^ and 90 cm source‐to‐surface distance (SSD). This field size is selected as an example where the field size is big enough to establish lateral charged particle equilibrium. OSL dosimeters were placed at the central axis (CAX) on both proximal and distal phantom surfaces so that both the entrance and exit dose could be measured. The dosimeters were also placed at CAX in air at 1.2 cm distance away from the phantom surfaces to get the in‐air readings. In the MC calculations, air was filled outside the solid water phantom.

**Figure 1:**
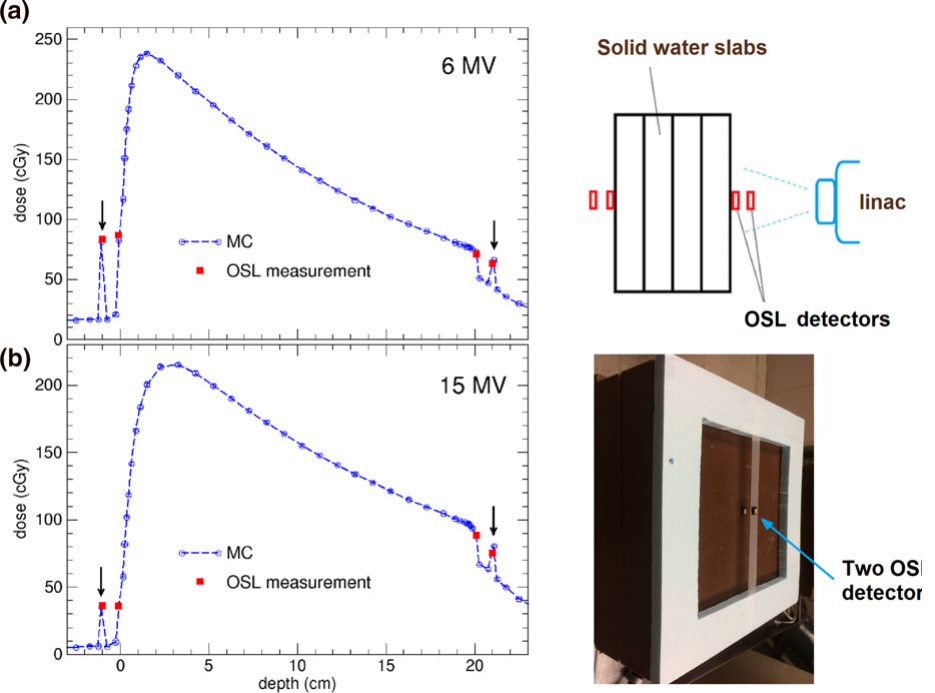
Comparisons of surface doses (entrance and exit) measured by OSL dosimeters and the doses calculated by Monte Carlo (MC) for (a) 6 MV, and (b) 15 MV photon beams of field size 4 × 4 cm^2^, delivered 200 MU on a water slab of 20 cm thickness. The arrows indicate the in‐air data points for which MC calculations include simulation of OSL dosimeter of size 10 × 10 × 1.5 mm^3^. The OSL measurement uncertainty is 3–5%, and the MC calculation statistical uncertainty is 1%. Also shown are schematic diagram and the photo of experimental arrangement. One OSL detector is on the thin tape in air and the other is on the solid water as shown in the photo insert.

Figure 1 shows the entrance/exit dose comparisons between MC and the measurements for the 6 MV and the 15 MV photon beams, together with the experimental layout. Similar results for 10 MV beam are observed and are not shown. To obtain absolute dose values for MC calculations, a calibration MC calculation is done with a 10 × 10 cm^2^ beam incident on a water phantom at 95 cm SSD. The dose at 5 cm depth is normalized to 100 cGy for 100 MU, corresponding to the linac output calibration of 1 cGy/MU for the TrueBeam linac. For MC calculations the results are consistent with the study by Devic et al. in their validation of MC for entrance dose calculations.^2^ MC calculation shows approximately 8% (~20 cGy out of 240 cGy maximum dose) entrance dose in air near proximal surface and 21% exit dose (~50 cGy out of 240 cGy) in air near distal surface for the 6 MV beam. For better comparison to OSL measurements, the OSL dosimeters are simulated in the MC calculations for data points where the measurements were taken, as indicated by the arrows in Figs. 1(a) and 1(b). The OSL dosimeter is modeled as a 10 × 10 × 1.5 mm^3^ water equivalent material, corresponding to the real size of the dosimeter, with a sensitive region of 5 × 5×0.2 mm^3^ inside. When the OSL dosimeter is included in the MC simulation, the MC calculated dosimeter dose is enhanced to about 82 cGy, very close to the OSL measurement of 85 cGy (~35% of maximum dose) for the 6 MV beam at the proximal surface and in‐air dose. The agreement between MC and OSL measurements shows the perturbation of the presence of nanoDot detector and demonstrates that the MC calculation is able to predict dose accurately under very complex geometry.

### 2.D Patient skin dose in real patient treatment plans

For CT image‐based patient dose calculations, the same patient CT images and the beam parameters, including jaw settings and MLC modulation sequences were used in MC simulations. Four materials (air, lung, tissue, bone) were employed in converting CT number to material and density. The MC calculated doses were calibrated to provide the same mean dose to the target as for Eclipse calculations. Three patient treatment plans were investigated which included different photon beam energy and treatment sites (H&N, lung, etc.). The treatment techniques that utilized 3D and VMAT were included in the evaluation.

**Figure 2:**
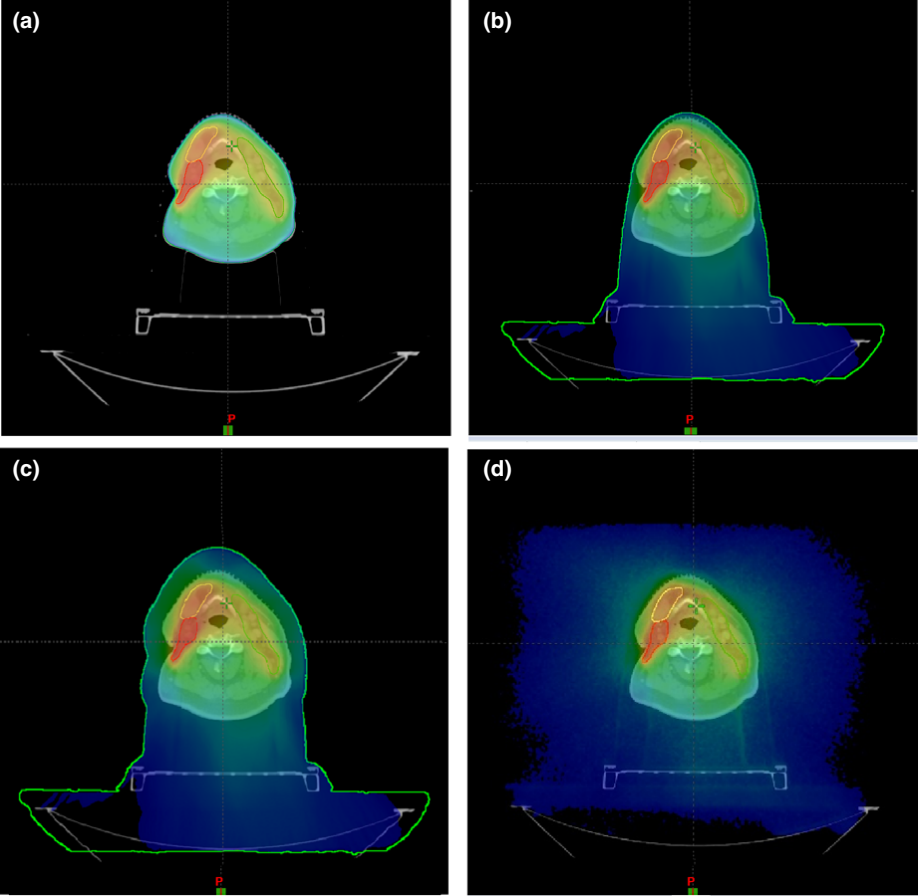
An axial view of patient comparison of dose distributions for an H&N cancer patient treated with 6 MV VMAT beams. (a) Eclipse with default body contour; (b) Eclipse with external body contour including head mask and table; (c) Eclipse with an extended external body contour including table; (d) Monte Carlo calculated with CT images including table.

**Figure 3:**
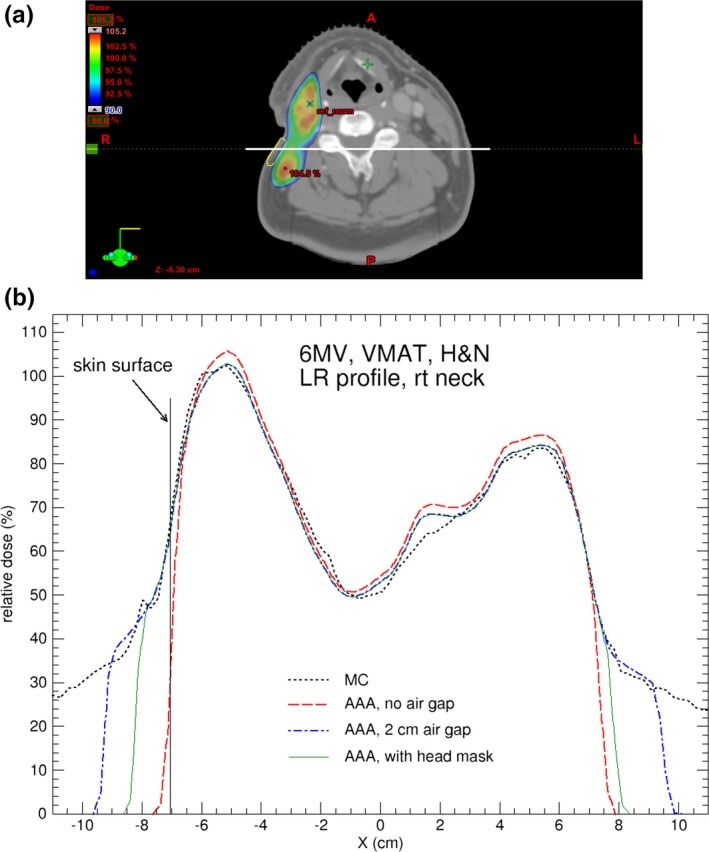
(a) Axial CT slice showing the locations of the contoured skin structure on right neck (in yellow) for the H&N cancer patient; (b) Comparison of dose profiles calculated by AAA and MC for the H&N case treated with 6 MV VMAT beams. The dose profile is indicated by the white horizontal line in (a). For AAA, the default body contour, 2 cm‐enlarged body contour, and body contour including head mask are used in the calculations. The MC calculation uncertainty is 1%.

**Figure 4:**
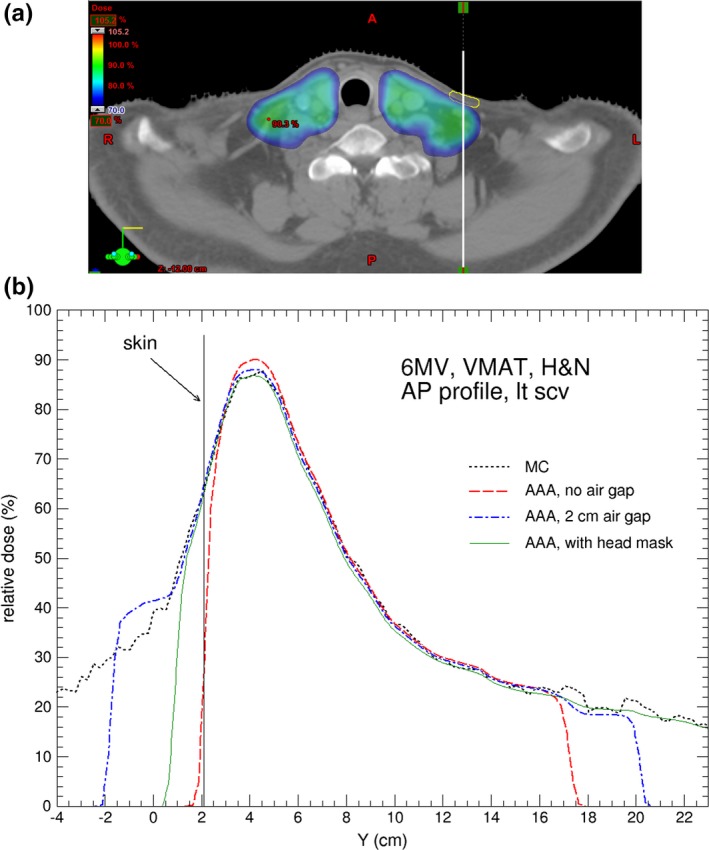
(a) Axial CT slice showing the locations of the contoured skin structure on left supraclavicular area (in yellow) for the H&N cancer patient; (b) Comparison of dose profiles calculated by AAA and MC for the H&N case. The dose profile is indicated by the white vertical line in (a). For AAA, the default body contour, 2 cm‐enlarged body contour, and body contour including head mask are used in the calculations. The MC calculation uncertainty is 1%.

The first case is a head‐and‐neck (H&N) cancer patient treated with 6 MV VMAT full arcs (Fig. 2). The default patient external body contour is constructed after CT images are imported into Eclipse. Dose calculations are performed only within this body contour, and no dose deposition outside of body contour [Fig. 2(a)]. In reality, there is always air present outside the patient's body, due to patient supporting/immobilization devices, such as a thermoplastic head mask. Immobilization devices can cause noteworthy effect on patient's skin doses,^6^, ^8^, ^9^ and to quantify the effect, the external body contour is extended to include the head mask and couch table [Fig. 2(b)]. Since it would be tedious task to make a detailed head mask contour, we simply extended a 2 cm air layer to the default body contour in addition to including the couch top within the body contour [Fig. 2(c)]. This can be done very easily with the margin tool in Eclipse. Including couch top in the body contour makes it appropriate for comparison with the MC simulation which automatically includes the couch top, though the difference is negligible from not including couch top (see below). It is worth noting that the calculated dose differences are resulting from the difference of external body contour as the same incident beams are used for all cases. The skin structures are contoured on the right neck and left supraclavicular area as shown in Figs. 3(a) and 4(a) (yellow contour lines). These locations are selected due to frequently seen severe skin reactions during H&N treatments.

Similar procedures are applied to the other two clinical cases, i.e., the same sets of Eclipse calculations are performed (except the calculation with contoured mask which only applies to H&N patients). In case #2 the target is a right lung tumor treated with 6 MV VMAT partial arcs. Two skin structures, anterior skin and right skin, are contoured at the respective locations of the body. In case #3 the treatment target is a rib treated with AP/PA opposing beams with 15 MV photon beams. One skin structure is contoured on the anterior chest.

## 3 RESULTS AND DISCUSSION

**Figure 5:**
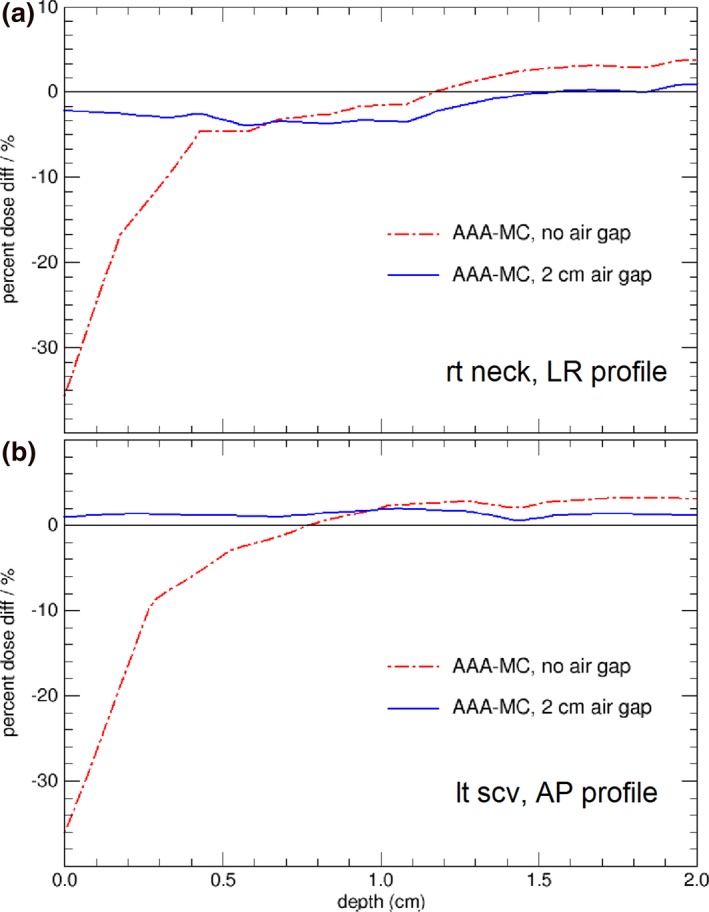
Dose difference between AAA and MC, with and without the air gap, from skin surface to 2 cm depth. (a) for the H&N dose profiles in Fig. 3(b); (b) for the H&N dose profiles in Fig. 4(b).

**Figure 6:**
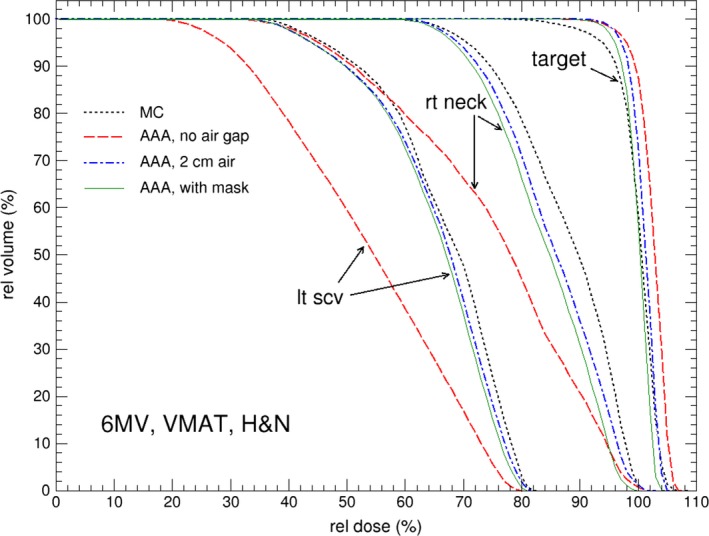
Comparison of dose volume histograms (DVH) for skin tissues and target for the H&N cancer patient, calculated from MC and AAA. For AAA, the default body contour, 2 cm‐enlarged body contour, and body contour including head mask are used in the calculations.

Figures 3(b) and 4(b) compare calculated dose profiles passing through the contoured skin structures for the H&N patient. The skin dose calculated by Eclipse with the head mask and table contoured [as in Fig. 2(b)] and with extended body contour [as in Fig. 2(c)] shows good agreement with the MC calculations. Figures 5(a) and 5(b) shows the dose difference near skin surface between AAA and MC for the respective dose profiles in Figs. 3(b) and 4(b). It is seen that the accuracy of skin dose calculation can be significantly improved by extending the body contour into air. This is one of the limitations of model based dose calculation algorithm which is inaccurate at the beam entry point. Skin dose is also evaluated by using dose volume histograms (DVH) for the contoured skin structures as shown in Fig. 6 where a comparison of DVH for the target and skin among the different external body contours tested. The choice of external body contours has a negligible effect (1–2%) on the target dose. This is expected, as the attenuation by the head mask and table is minor. Although the choice of external body contour has little effect on the target dose in all calculations, it has considerable effect on the accuracy of skin dose. It is also seen that, for Eclipse, there is no noticeable difference on skin dose between the body contour with the head mask and the body contour simply extended by 2 cm; both are closer to MC calculated skin dose.

Table 1 lists the relative mean dose calculated by MC and Eclipse of different external body contour scenarios for the H&N cancer patient. The results show Eclipse underestimates skin doses with default body contour by more than 14% of prescription dose. Substantial improvement is observed when the external body contour is extended to include head mask or air. The difference between MC and Eclipse calculated mean skin doses agrees within with 4%. In addition, the difference in calculated mean skin doses is less than 0.5% between extended body contour by 2 cm and detailed head mask contour in Eclipse calculations. In fact, we also performed Eclipse calculations with body contour extended by 1 cm and found no major difference on the calculated skin dose compared to 2 cm extension. This result demonstrates that in practice Eclipse skin dose calculation accuracy can be appreciably improved by simply extending the external body contour by at least 1 cm. The main reason that this method is able to increase the skin dose calculation accuracy is by moving the entry point away from skin since the inaccuracy occurs at entry point for the model based calculation algorithm. This simple extension technique achieves the result by including the skin masks in the study of comparing static IMRT and VMAT delivery techniques.^19^

**Table 1:** Mean percentage (%) skin dose and target dose for the H&N cancer case. Calculated by MC and Eclipse AAA of different scenarios (see text for details). The MC calculation uncertainty is 1%

	AAA, default	AAA, 2 cm air, w/tbl	AAA, mask w/tbl	MC
Right neck	75.1	83.9	83.9	87.6
Left scv	53.7	64.7	64.9	66.8
PTV	102.5	99.9	100	100

Table 2 compares the relative mean skin doses calculated by MC and Eclipse (with and without body contour extension of 2 cm) for the three cases investigated. Note that here the extended body contour does not include the couch top, i.e., it is simply extended by 2 cm from the default body contour. For the H&N case with 2 cm extended contour, compared to the data in Table 1, it only has a roughly 1% difference on skin dose, with or without couch in body contour. In general, based on results in Table 2, Eclipse underestimated skin dose by up to 14% of target dose when default external body contour is utilized. The accuracy of Eclipse predicted skin dose improves when the body contour was extended by 2 cm outside the skin and the patient skin dose predicted by Eclipse are within 4% of the target dose from the MC calculated mean skin dose.

**Table 2:** Mean percentage (%) skin dose for all the three cases. Calculated by MC and Eclipse AAA of different scenarios (see text for details). The MC calculation uncertainty is 1%

	Skin location	AAA, default	AAA, 2 cm air	MC
15 MV rib	Anterior	64.5	78.9	75.1
6 MV lung	Anterior	33.8	40.2	39.0
	Right	20.2	24.8	24.8
6 MV H&N	Right neck	75.1	85.2	87.6
	Left scv	53.7	65.5	66.8

## 4 CONCLUSIONS

This study has shown that Eclipse TPS generally underestimated skin doses by up to 14% of prescription dose depending on how external body is contoured. The largest underestimation occurs when the external body contour started at the patient's skin. It is known that the model based dose calculation has limitations especially at the calculation entry point. By moving the calculation entry point away from the skin it effectively improves the calculation accuracy at the skin. We have shown that the underestimation of skin dose in Eclipse can be considerably improved by extending external body contour by at least 1 cm to include a portion of air outside of the skin. This method is applicable to Eclipse treatment planning system with AAA calculation algorithm. When utilizing immobilization masks or body casts, extending the external body contour from the skin would automatically include the masks and make the accuracy of skin dose calculations within 4%. Dose delivered to deeper target volumes or organs at risk are not affected. This maneuver, then, can substantially reduce predicted error of skin dose; this is crucial for accurate expectation of toxicity by the clinician.

## CONFLICT OF INTEREST

The authors have no conflict of interest to disclose.
